# Do Genes Play a Role in the Decoy Effect?

**DOI:** 10.3389/fpsyg.2020.523299

**Published:** 2020-10-20

**Authors:** Jianmin Zeng, Xinyi Zhao, Huihui Qin, Xingrong Hou, Qinglin Zhang

**Affiliations:** Sino-Britain Centre for Cognition and Ageing Research, Faculty of Psychology, Southwest University, Chongqing, China

**Keywords:** decoy effect, behavioral economics, behavioral genetics, neuroeconomics, decision making

## Abstract

The decoy effect arises when the ratio of choosing B from A and B options is lower than the ratio of choosing B from A, B, and D options, wherein D is dominated by B. This decision pattern is obviously unreasonable but quite common. Previous research suggested that impulsive people have stronger decoy effect. Rs806379, as a single-nucleotide polymorphism (SNP) locus of cannabis receptor 1 gene (*CNR1*), has significant effect on impulsivity—people of A/A genotype are more impulsive than others. Therefore, rs806379 may relate to the decoy effect, which was tested in this study. Participants (359 Han Chinese college students) finished a task of the decoy effect, in which they made decisions between two or three mobile hard disks with various prices and provided saliva for genotyping. The results revealed the existence of the decoy effect. Furthermore, we found that participants with A/A genotype (251 Han Chinese college students) showed stronger decoy effect than others, when the prices were not high. This is the first attempt to study the decoy effect from a gene perspective. The result shows that even an SNP of a gene can have a significant association with complex human economic decision-making activities.

## Introduction

Imagine that a group of people choose between two options A and B, and the ratio of choosing B is *x*%. At a separate time point, this same group of people choose among three options A, B, and D, wherein D is dominated by B (D is worse than B in at least one aspect, and D is the same as B in all other aspects), and the ratio of choosing B is *y*%. If people are rational, then *y* should be no larger than *x* for the following reasons. Given that B dominates D, if an individual prefers A to B in the two-option condition, then he should prefer A to the other options in the three-option condition; if an individual prefers B to A in the two-option condition, then he should prefer B to the other options in the three-option condition. Taken together, the ratio of choosing B (*y*%) in the three-option condition should be no larger than that in the two-option condition (*x*%). Nevertheless, people often get *y* being larger than *x*. This irrational decision phenomenon is called the decoy effect.

In other words, the decoy effect is the phenomenon that the attraction of B (relative to A) can be boosted by adding an additional option D, which is dominated by B (Li et al., 2019). It is also called asymmetric-dominance effect or attraction effect (Huber and Mccann, 1982). The decoy effect is one of the most robust findings in the area of decision-making. It has been demonstrated in many fields, such as medical decisions (Schwartz and Chapman, 1999), consumer choices, gamble preferences, and so on (Huber et al., 1982; Heath and Chatterjee, 1995).

Some research suggested that decoy effect is related to impulsivity (Kowal and Faulkner, 2016). This is comprehensible. Imagine a person faces two options A and B. He has to compare them to reach a final decision. Now imagine at a separate time point he faces three options A, B, and D, wherein D is dominated by B. Given that B dominates D, this person, if being impulsive, would like to immediately choose B and spend little effort in comparing A and B, which results in decoy effect.

The cannabis receptor 1 (CB1) is a well-characterized cannabinoid receptor that mediates endogenous cannabinoid signaling (Matsuda et al., 1990). It is also widely distributed in brain regions related to drug reward and drug memory, including the hippocampus, striatum, and cerebral cortex (Herkenham et al., 1990; Matsuda et al., 1990; Tsou et al., 1998). In a sample of Southwest Californian Indians, Ehlers reported a significant association between polymorphisms of the CB1 receptor encoding gene *CNR1* and impulsivity (Ehlers et al., 2007).

Rs806379 is a single-nucleotide polymorphism (SNP) of *CNR1* and is in intron 2 of *CNR1*. The A/A allele in rs806379 was positively correlated with impulsivity (Ehlers et al., 2007). Some study also found that the rs806379 homozygous carriers (A/A carriers) were linked to enhanced impulsivity (Buchmann et al., 2015). Each additional A allele would lead to an increase in impulse control problems; that is to say, A allele has a substantial association with impulsivity (Buchmann et al., 2015).

Taken together, A/A genotype is related to impulsivity, and impulsivity was reported to be related to the decoy effect. Therefore, we hypothesized that people with A/A allele would exhibit stronger decoy effect than others.

## Materials and Methods

### Participants

In total, 368 healthy undergraduates (256 females) both completed a consumption-decision task and were successfully genotyped for the *rs806379*. Nine of them were excluded from analysis (see section “Results” for details). Therefore, we finally had 359 participants (249 females). They all provided written informed consent and got paid for their participation. The university is located in China, and all participants were Han Chinese. These participants’ ages ranged from 17 to 23 years (average = 19.87 years; standard deviation = 1.01 years). They were all right-handed. None of them had a history of psychological or neurological disorders, or drug/alcohol/smoking problems. This study was approved by the local ethical committee (see Ethics Statement). The payment was not associated with task performance or decisions they had made for the following reasons: (1) it is hard for us to ask the subjects to pay money for the disks; (2) if we paid subjects according to their performance scores, then it could distort decisions of participants, especially late participants. Given that we had so many subjects, it is very hard for us to ensure that the early subjects never told some of late subjects how to earn more money. Therefore, we did not associate payment with their decisions, but we instructed them to respond to the questions as in reality. All subjects got equal payment in the premise that they finished the experiment. The gene samples were collected after participants finished the experimental tasks.

### Genotyping

Saliva was collected from each participant for DNA extraction using the Mass Array System (Agena iPLEX Assay, San Diego, CA, United States). First, we isolate approximately 10–20 ng of genomic DNA from the saliva samples. Polymerase chain reaction (PCR) amplification was conducted using the following primers: ACGTTGGATGGACTTACTTTTGTGTCAGGC and ACGTTGGATGTGCCTAAATCGCAGAACTGA. The sample DNA was amplified by a multiplex PCR reaction, and then the obtained products were used for locus-specific single-base extension reaction. Unextended primers used in the study were GAACTGATCTGAAATTAGATGA. At last, the resulting products were desalted and transferred to a SpectroCHIP array. The alleles were discriminated by mass spectrometry (Agena). *Rs806379* genotype was coded as a categorical variable (A/A, A/T and T/T) for the subsequent analysis. Hardy–Weinberg equilibrium was tested. See section “Genotype Frequencies” for details.

## Experimental Task

**FIGURE 1:**
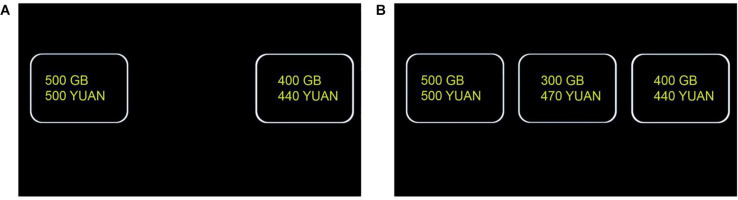
A trial in the baseline condition **(A)** and a trial in the decoy condition **(B)**.

This experiment contained two within-subject conditions: baseline condition and decoy condition. To prevent the subjects from seeing through the trick of this kind of task, we took three measures: (1) these two conditions were administered separately as two sessions; (2) decoy session went first, and the baseline session went second, because the baseline-condition trials were relatively simple (Figure 1) and could be more easily memorized; (3) we arranged the subjects to do irrelevant tasks (>10 min) between these two sessions.

Each session contained an instruction and some decision trials. At the end of the instruction, the participants could choose to move on or to reread the instruction. This design was to increase the possibility that the participants really understood the task before doing the decision trials.

In the baseline condition, each trial contained two options (mobile hard disks): A and B. A options were composed in the following way: five memories (300, 400, 500, 600, 700 GB) ^∗^ five prices (300, 400, 500, 600, 700 Yuan). This defined 25 trials. B’s memory = A’s memory−100. B’s price was such that A’s memory–price ratio was always slightly higher than B’s. See Supplementary Table 1 for details. The positions (left and right) were counterbalanced for A and B. All participants saw all 25 combinations (trials), which were presented randomly, except that the combination (trial) of 500 GB—500 Yuan always went first so that all subjects had the same initial memory and price, which might serve as a reference for late trials.

In the decoy condition, the procedure was the same as that in the control condition except the following aspects. Each trial contained three options: A, B, and D (decoy option). A and B were identical as those in the baseline condition. D was dominated by B (but not by A): D was worse than B in all dimensions; i.e., D had smaller memory than B and was more expensive than B. The positions (left, middle, and right) were counterbalanced for A, B, and D.

Figure 1 presents exemplar trials of the baseline and decoy conditions.

## Results

### Genotype Frequencies

Among 368 Han Chinese participants, 251 were A/A allele carriers, 108 were A/T allele carriers, and 9 were T/T allele carriers. The genotype frequencies did not deviate from Hardy–Weinberg equilibrium (χ^2^ = 0.43, *P* > 0.05). Totally, the minor allele frequency was 0.17, which is much lower than that for African (0.56), European (0.45), and American (0.33), but is consistent with that for East Asian (0.21) reported by 1000Genomes^1^. Considering that the sample of T/T genotype (*n* = 9) in this study was too small to represent the population of this genotype, we omitted these T/T carriers from the further analysis. That is, we have 359 participants for data analysis.

### Behavioral Results

A trial in the baseline condition and a trial in the decoy condition, if having the same A and B options, constituted a pair. For each pair, following the definition of decoy effect, we calculated decoy effect as follows. If a subject chose A in the baseline condition but chose B in the decoy condition, then the score of decoy effect is 1; if a subject chose B in the baseline condition but chose A in the decoy condition, then the score is −1; otherwise, the score is 0.

**FIGURE 2:**
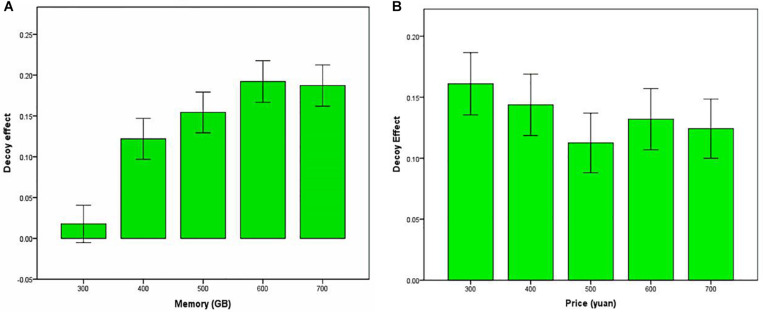
The decoy effects at different memories **(A)** and prices **(B)**. Error bars: 95% confidence intervals.

Figure 2 presents the decoy effects at different prices and memories. All of them are larger than 0, indicating the existence of decoy effect. We performed a repeated-measures full-factorial analysis of variance (ANOVA), with the dependent variable being the score of decoy effect, and the independent variables being price and memory. Greenhouse–Geisser correction was applied wherever necessary. The main effect of memory was significant: *F*(4,1432) = 30.52, *P* < 0.001. The main effect of price was also significant: *F*(4,1432) = 2.71, *P* < 0.05. The interaction between memory and price was significant: *F*(16,5728) = 4.98, *P* < 0.001.

### The Influence of *rs806379* on Decoy Effect

**FIGURE 3:**
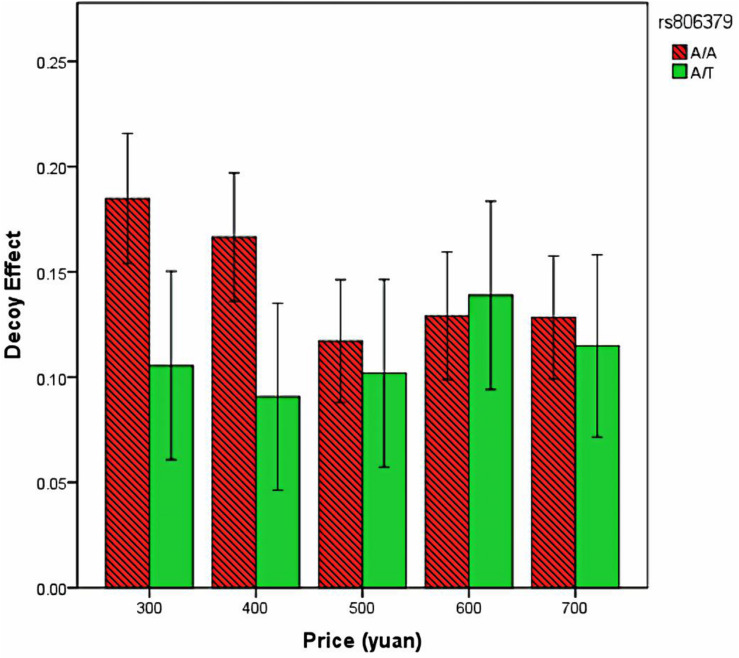
The decoy effect for participants with different genotypes. Error bars: 95% confidence intervals.

We performed a repeated-measures full-factorial ANOVA with the dependent variables being the decoy effect and the independent variables being the price, memory, and genotype. Greenhouse–Geisser correction was used when necessary. Although the main effect of genotype was not significant: *F*(1,357) = 1.79, *P* > 0.05, the interaction between genotype and price was significant: *F*(4,1428) = 2.69, *P* < 0.05. Figure 3 showed that the gene effect on decoy effect was large when the prices were not high. The interaction between genotype and memory was not significant: *F*(4,1428) = 0.13, *P* > 0.05. The interaction between genotype, price, and memory was not significant: *F*(16,5712) = 0.55, *P* > 0.05. To exclude the possible influence of gender on the effects, we further added gender (coded as 0 and 1) as covariates into the above ANOVA. Again, we found a significant interaction between genotype and price: *F*(4,1424) = 2.73, *P* < 0.05. Other main or interaction effects involving genotype were all nonsignificant.

## Discussion

In our behavior results, we repeated the decoy effect. Adding a decoy option enhanced subjects’ preference for the target option B. This is consistent with the assertion that the decoy effect is a stable phenomenon (Huber and Mccann, 1982). We hypothesized that the decoy effect can be affected by *CNR1* rs806379. This hypothesis was supported by the gene results: Participants with A/A genotype showed stronger decoy effect than others, when the prices were not high.

The effect of rs806379 polymorphism on decoy effect is novel but comprehensible. First, *CNR1* rs806379 polymorphism can affect impulsivity. Participants with A/A genotype showed stronger impulsivity than those with A/T genotype (Buchmann et al., 2015). Second, impulsivity is connected with decoy effect (Kowal and Faulkner, 2016). Therefore, compared with others, A/A carriers had stronger decoy effect, when the prices were not high. However, when prices became high, this gene effect on decoy effect disappeared. This is possibly because when the prices were high, the decision situation became more serious, and thus A/A carriers became more careful and no longer too impulsive and thus behaved like others. In addition, both genotypes exhibited decoy effect at some degree. This might be explained as all people have some degree of impulsivity, because all people have impulsive system 2 as a part of their brain (Kahneman, 2011).

This study has several limitations, which can be the directions for future research. This study used only Chinese subjects. What will happen if researchers use Western subjects? Given that Western subjects differ from Chinese subjects in both genes and culture, this question should be quite interesting. Are there other genes relating to the decoy effect? Most of participants in this study were females. Although the main conclusion still stood after excluding the influence of the gender, a gender-balanced study should be a better choice. Participants in this study could reread the instruction, which could improve but could not ensure their understanding of the instruction. This study used mobile hard disks as experimental material, which might induce different responses from people with different attitudes toward mobile hard disks. Using various commodities is recommended for the future research. In this study, the decoy condition had more visual load than the control condition. Although this practice is common in studies about the decoy effect, this can still cause worry about this potential confounding factor. Although it is hard to imagine how this confounding factor encourages people to choose the target option rather than other options, a study on decoy effect will be perfect if it can exclude this confounding factor. This article interprets the genotype’s effect on the decoy effect as impulsivity, but does not provide strong support for this interpretation. Future research can design special experiments to test this interpretation against other interpretations.

As far as we know, this study provides the first direct evidence for the possible contribution of genes to decoy effects. Our finding shows that human innate factors even like SNPs can possibly affect complex economic decision-making activities.

## Data Availability Statement

The raw data supporting the conclusions of this article will be made available by the authors, without undue reservation, to any qualified researcher.

## Ethics Statement

The study involving human participants was reviewed and approved by the Administrative Committee of Psychological Research at Southwest University. The patients/participants provided their written informed consent to participate in this study.

## Author Contributions

All authors conceived and designed the experiments, collected and analyzed the data, and wrote and revised the manuscript.

## Conflict of Interest

The authors declare that the research was conducted in the absence of any commercial or financial relationships that could be construed as a potential conflict of interest.

## Footnotes

1 https://www.ncbi.nlm.nih.gov/snp/rs806379#frequency_tab. Retrieved on August 19, 2020.
