# Supplementary material for: Do Genes Play a Role in the Decoy Effect?
Source: Front Psychol. 2020 Oct 20;11:523299. doi: 10.3389/fpsyg.2020.523299 (PMC7606847; doi:10.3389/fpsyg.2020.523299)
Supplement: Supplementary file 1 [file Table_1.DOCX]

| Table S1. Memories and prices. | | | |
| --- | --- | --- | --- |
| A memory | A price | B memory | B price |
| 300 | 300 | 200 | 220 |
| 300 | 400 | 200 | 290 |
| 300 | 500 | 200 | 370 |
| 300 | 600 | 200 | 440 |
| 300 | 700 | 200 | 510 |
| 400 | 300 | 300 | 250 |
| 400 | 400 | 300 | 330 |
| 400 | 500 | 300 | 410 |
| 400 | 600 | 300 | 500 |
| 400 | 700 | 300 | 580 |
| 500 | 300 | 400 | 260 |
| 500 | 400 | 400 | 350 |
| 500 | 500 | 400 | 440 |
| 500 | 600 | 400 | 530 |
| 500 | 700 | 400 | 620 |
| 600 | 300 | 500 | 280 |
| 600 | 400 | 500 | 370 |
| 600 | 500 | 500 | 460 |
| 600 | 600 | 500 | 550 |
| 600 | 700 | 500 | 640 |
| 700 | 300 | 600 | 280 |
| 700 | 400 | 600 | 380 |
| 700 | 500 | 600 | 470 |
| 700 | 600 | 600 | 570 |
| 700 | 700 | 600 | 660 |
